# The effect of a self-help book for shift work disorder – a randomized controlled trial

**DOI:** 10.3389/fpsyt.2026.1829148

**Published:** 2026-08-26

**Authors:** Siri Waage, Anette Harris, Øystein Vedaa, Ståle Pallesen, Erlend Sunde, Bjørn Bjorvatn

**Affiliations:** 1 Norwegian Competence Center for Sleep Disorders, Haukeland University Hospital, Bergen, Norway; 2 Department of Global Public Health and Primary Care, University of Bergen, Bergen, Norway; 3 Department of Psychosocial Science, University of Bergen, Bergen, Norway; 4 Department of Health Promotion, Norwegian Institute of Public Health, Bergen, Norway; 5 Solli District Psychiatric Hospital, Bergen, Norway

**Keywords:** health care workers, RCT, self-help therapy, shift work, shift work disorder

## Abstract

**Introduction:**

This randomized controlled trial (NCT05633498) compared the effects of a self-help book for coping with shift work (intervention) with an active control condition consisting of sleep hygiene advice among healthcare workers.

**Methods:**

Data were collected from 199 healthcare workers (100 in the intervention group and 99 in the control group) with shift work disorder (SWD) (86.9% females, mean age 39 years). Participants completed questionnaires on SWD and sleep, a knowledge test about shift work, turnover intention, self-reported sickness absence and work-related fatigue at baseline and 6 months follow-up. Linear mixed-effects models were used for continuous outcomes, mixed-effects logistic regression for binary outcomes, and mixed-effects negative-binomial regression for count outcomes.

**Results:**

In total, 136 participants (68.3%) completed follow-up. SWD prevalence decreased to 83.1% in the control group and 79.0% in the intervention group (no significant interaction effect). Knowledge scores increased in the intervention (7.5 to 8.4), but not in control (7.2 to 7.1). No significant interaction effects were found for turnover intention (estimate = -0.64, SE = 0.42, p = 0.13), sickness absence (IRR = 0.85; 95% CI: 0.44–1.62; p = 0.62), or sleep related sickness absence (IRR = 0.59; 95% CI: 0.24–1.43; p = 0.24). For three of five fatigue dimensions; lack of energy (p = 0.014), lack of motivation (p = 0.018), and sleepiness (p = 0.021), the time × group interactions were statistically significant favoring the intervention.

**Conclusion:**

Compared with sleep hygiene advice, the self-help book was associated with greater improvements in knowledge about shift work and three dimensions of self-reported fatigue. However, it did not reduce SWD or insomnia, and the findings do not support the book as an effective treatment for SWD.

**Clinical Trial Registration:**

https://clinicaltrials.gov/study/NCT05633498?cond=Shift%20Work%20Disorder&viewType=Card&rank=6, identifier NCT05633498.

## Introduction

Shift work may disrupt biological rhythms and is associated with several negative health outcomes, in which sleep problems are among the most commonly reported complaints (1). As linked to negative health, shift work is also associated with sickness absence (2). In Europe, more than one in three workers are engaged in nonstandard work schedules (3), and approximately 19% of the work force report to be engaged in night work at least once a month (4). Nurses and other health care workers are often involved in rotating shift work involving both night work and quick returns (QRs), the latter defined as a rest period between two consecutive shifts of less than 11 hours (5, 6).

Shift work disorder (SWD) is a circadian rhythm sleep-wake disorder characterized by symptoms of insomnia during the sleep period and/or excessive sleepiness during the wake period due to work hours that overlap with the habitual time for sleep and which lead to a reduction in sleep duration (7–9). A systematic review and meta-analysis estimated the overall prevalence of SWD to be 26.5% (10). Among nurses, the reported prevalence varies by work schedule, ranging from 4.8% in day workers to 44.3% in those working three-shift rotations (11). SWD is associated with considerable adverse consequences for the individual, employers and the society (12). At the individual level, SWD has been associated with impaired health, with affected workers reporting more health complaints and sleepiness-related accidents compared to workers without SWD (13–15). Further, at the societal level, SWD is linked to increased turnover intention among nurses (16). As turnover intention is shown to be a strong predictor for actual turnover, this could potentially lead to reduced clinical care and increased economic costs for health institutions (17). Thus, the consequences of SWD may lead to increased service costs and lower standards of care (18).

Treatment approaches targeting the core features of SWD include managing sleep and sleepiness problems related to the work schedule, as well as to the circadian rhythm sleep-wake disorder itself. Recommendations addressing problems related to the circadian rhythm include use of bright light and melatonin, with the timing of both being crucial for treatment efficacy. Additionally, education about sleep hygiene, circadian rhythms, and the importance of prioritizing sleep are also recommended (14, 19). As insomnia often is a core symptom of SWD, recommended treatment addressing sleep and sleepiness problems includes both non-pharmacological strategies and pharmacological interventions in the form of hypnotic medication (20).

Self-help treatments have been proposed as an inexpensive and easily accessible alternative to face-to-face non-pharmacological treatments for insomnia (21, 22). Although the meta-analysis by van Straten and Cuijpers reported small to moderate effect sizes, self-help treatments, when integrated in a stepped care approach, were considered to constitute a useful addition to existing treatment options for insomnia (21). Bibliotherapy, implying treatment administered through written material, is described as an innovative and effective treatment in individuals with insomnia (23). For instance, a recent meta-analysis reported large effect sizes for cognitive behavioral therapy for insomnia (CBTi) through guided bibliotherapy (24). Sleep hygiene refers to recommendations for optimizing sleep. While such recommendations can improve sleep, they are normally not considered sufficient and effective for chronic insomnia (22, 25). It has previously been shown that a self-help book for insomnia, emphasizing CBTi, significantly improved several sleep parameters compared to sleep hygiene advice among patients with chronic insomnia (26). However, due to the high prevalence of SWD among shift workers, it is important to address insomnia symptoms specifically related to the work schedule. Accordingly, the aim of the present study was to compare the effects of two written materials, a self-help book for coping with shift work versus a leaflet containing basic sleep hygiene advice, in a randomized controlled trial. We hypothesized that:

H1: A self-help book for coping with shift work will reduce the prevalence of SWD and reduce sleep- and sleepiness problems more than sleep hygiene advice.
H2: A self-help book for coping with shift work will increase knowledge about sleep and circadian rhythms related to shift work more than sleep hygiene advice.
H3: A self-help book for coping with shift work will reduce turnover intention, sickness absence and fatigue more than sleep hygiene advice.

## Materials and methods

**Figure 1:**
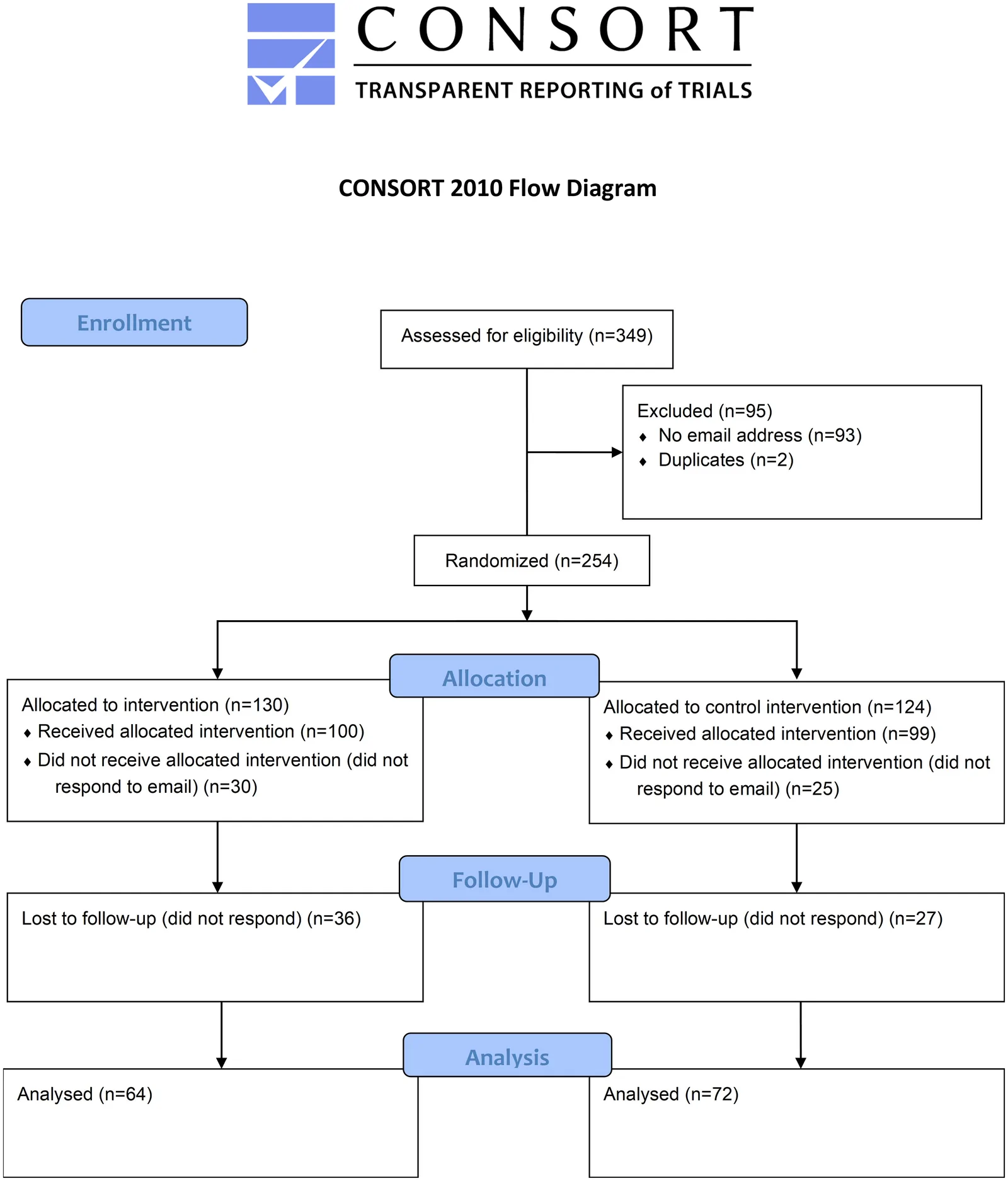
CONSORT 2010 flow diagram of the study.

The goal was to recruit 300 participants to a randomized controlled trial to compare two types of written material as treatments for SWD: 1) The self-help book entitled “Shift work and sleep. How to cope with night work and irregular work hours” (English translation), and 2) standard sleep hygiene advice (active control) provided on a one-page leaflet (**Table 1**). The self-help book was written specifically for shift workers struggling with night- and shift work and consists of three parts: 1) information about sleep regulation and circadian rhythms, 2) potential consequences of shift work on health and, 3) specific recommendations about how to cope with different types of shift work. The data collection took place from Spring 2023 to Autumn 2024. Initially, healthcare workers were recruited through invitations posted on the internal website and wall posters at two hospitals in the Western region of Norway, with the heading “Do you want to cope better with shift work?”. The health care workers were informed that the study compared two types of written material to better cope with shift work, but they were not given any details about what type of material. The invitation further included information about the project and a link to a questionnaire that was administered by the web-based survey platform SurveyXact (by Rambøll). Healthcare workers who fulfilled the criteria for SWD by endorsing three items reflecting the diagnostic criteria were invited to consent to participate in the study and were granted access to the additional questions. Healthcare workers not fulfilling the criteria were not invited to the study. To ensure enough participants, a group of nurses already participating in the ongoing Survey of Shift Work, Sleep and Health (SUSSH) were invited to participate. They received an email with the same invitation and information that was published on the hospital website. More information about SUSSH is available in several publications (11, 27, 28). Finally, a total of 349 healthcare workers consented to participate. Of those, 93 did not provide their contact information, and two were duplicates, leaving 254 individuals to be randomized to the two groups of treatment. Randomization was conducted using a computer-generated allocation sequence produced by a random list team generator (https://www.randomlists.com/team-generator). The randomization list was created prior to participant enrollment by the principal investigator. Participants were assigned to the intervention or control group in the order in which they were enrolled. The authors performing the statistics were blinded to group affiliation until the statistical analyses were conducted. Due to lack of response to the email about how to receive the study material, a total of 199 workers received material containing either the self-help book (n=100) or the sleep hygiene advice (n=99). After six months, 63 participants (27 in the sleep hygiene group and 36 in the self-help book group) did not complete the follow-up survey, amounting to a response rate of 68.3%. Upon completion of the follow-up, participants were offered the written material they had not received at baseline. **Figure 1** displays an overview of the participant flow. The study was approved by the Regional Committee for Medical Research Ethics, Western Norway (372901), and was preregistered in Clinical Trials (NCT05633498).

**Table 1:** The sleep hygiene advice used as control condition.

Avoid caffeinated drinks during the last hours before bedtime (coffee, tea, cola).
Avoid smoking/nicotine during the last hours before bedtime.
Avoid alcohol as a sleep aid.
Avoid going to bed hungry, but do not consume a heavy meal before bedtime.
Keep the bedroom dark, quiet, and with moderate temperature. If necessary, use mask and earplugs.
Regular exercise is good, but do not exercise during the last hours before bedtime.

## Instruments

### Demographics and other variables

The questionnaire comprised questions about sociodemographic variables like age, sex and marital status. In addition, the questionnaire included questions about work schedule (day only, evening only, two-shift including day and evening, night only, three-shift schedule including day, evening and night, and other schedules including night work). Use of sleep medication during the last month was assessed both in terms of prescribed and over the counter medications. At follow-up, the participants were additionally asked three questions about the written material included in the study: “Have you read the written material?”, with response alternatives “no”, “yes parts of” of “yes all material”. In addition, the workers answered the statement “I am satisfied with the written material I received?” with 5 response alternatives “strongly disagree”, “disagree”, “neither agree nor disagree”, “agree” and “strongly agree”. The last item was “I have followed the advice from the written material I received” with the same 5 response alternatives.

### Shift work disorder

SWD was assessed with three previous used (28) questions based on the criteria in the ICSD-3 (9): a) Do you have a work schedule that sometimes overlaps with the time you usually sleep?, b) if yes, does this cause insomnia and/or excessive sleepiness due to reduced amount of sleep?, c) if yes, has this lasted for at least three months? Participants were classified as having SWD when endorsing all three questions.

### Sleep and sleepiness

Symptoms of insomnia and sleepiness related to day-, evening-, night shifts and rest-days respectively, were measured with the Bergen Shift Work Sleep Questionnaire (BSWSQ) (29). The questionnaire includes seven questions where each item is rated on a 5-point scale, ranging from 0 to 4 (“never,” “rarely,” “sometimes,” “often,” and “always”). The reported frequency indicates the persistence of each symptom, associated with day-, evening-, night-shifts or rest days, respectively. Symptoms reported as occurring “often” or “always,” indicate more severe sleep or sleepiness problems. Four context-specific insomnia composite scores (day shift, evening shift, night shift, and rest days) were calculated by adding separate scores, ranging from 0 to 24 for each shift. For each shift type, participants were classified as not having or having shift-related insomnia. In the present study the Cronbach’s alpha was.88.

### Knowledge test about sleep, health and shift work

We used 13 previous designed questions to measure knowledge about how to prevent negative consequences of shift work, published in Norwegian (30). The questions varied in terms of topic, ranging from sleep–wake rhythms to characteristics of sleep after night shifts. For each question the participant should evaluate four statements and select the one that best represents the correct answer. For instance, “Which statement is most correct?” with the following statements; 1) “Younger people generally seem to cope with night work worse than older people.”, 2) “Age has no impact on how people cope with night work.”, 3) “Younger people generally seem to cope with night work better than older people” or 4) People between 40 and 50 cope best with night work.”. Another statement was “Shift work disorder has the following diagnostic criteria, except?” with the following statements; 1) “Complaints of insomnia or excessive sleepiness.”, 2) “Insomnia or sleepiness can be related to the work schedule.”, 3) “Low mood after night work.”, or 4) “Symptoms must have lasted for more than three months.”. One final example of a statement was “What characterizes sleep after a night shift (compared to normal night time sleep)?” with the following statements; 1) “Longer time than usual to fall asleep.”, 2) “Shorter sleep duration.”, 3) “Increase of REM sleep.”, or 4) “More light sleep.”. Each correct statement was given one point, and the scores were summarized to a composite score of correct answers, with a maximum of 13 points.

### Turnover intention

Turnover intention was measured with the Turnover Intention Scale (TIS) which is comprised of three items adapted from Michigan Organizational Assessment Questionnaire (31). The three items are: “I will actively look for a new job in the next year.” “I often think about quitting.”, and “I will probably look for a new job by the next year.” Responses were recorded on a 5-point Likert scale from 1 (strongly disagree) to 5 (strongly agree), an aggregated into a composite score ranging between 3 and 15. A high score indicates a high degree of turnover intention. Cronbach’s alphas were.92 at baseline and.93 at follow-up.

### Self-reported sickness absence

Two self-report questions about sickness absence were included; “How many days have you been absent from work in the past 3 months due to illness?” and “Can you estimate how many of those days of absence were caused by shift work-related sleep problems?”. Response alternatives for both questions were a drop-down menu ranging from “0” to “90” days.

### Swedish occupational fatigue inventory

Work related fatigue was assessed with the Swedish Occupational Fatigue Inventory (SOFI) (32). Participants were asked to indicate the extent to which they had experienced a list of 20 psychological and physical sensations related to fatigue the last week. Five dimensions of fatigue were calculated, each subscale representing a distinct aspect of fatigue; *lack of energy, physical exertion, physical discomfort, lack of motivation* and *sleepiness*. Each dimension has a composite score ranging between 0 and 6. Cronbach’s alphas were: *Lack of energy:* α = .87 and α = .92; *physical exertion:* α = .70 and α = .75; *physical discomfort:* α = .73 and α = .80; *lack of motivation:* α = .81 and α = .88; and *sleepiness:* α = .76 and α = .84 for baseline and follow-up, respectively.

## Statistical analysis

Power analysis was conducted with G*Power, version 3.1.9.2 (33). Expecting a small effect size (Cohens d=0.20), with an alpha of.05 (two-tailed), power (1-β) of.80, and a correlation coefficient for repeated measures of.50, it was determined that a total sample size of 164 was needed to detect significant group by time interaction effects.

Descriptive analyses and McNemar and paired t-tests were used to compare values before and after the intervention within each condition, utilizing IBM SPSS, version 29.0.2.0. All analyses related to time x group effects were conducted in R, version 4.4.1 (2024–06–14). Statistical significance was set at α = 0.05 (two-tailed). Continuous longitudinal outcomes were analyzed with linear mixed-effects models, binary outcomes with mixed-effects logistic regression, and count outcomes with mixed-effects negative-binomial regression. For every mixed model, a random intercept for participants was specified to account for intra-individual correlation arising from repeated measurements at baseline and follow-up. Fixed effects comprised group (0 = control, 1 = intervention), time (0 = baseline, 1 = follow-up), and their interaction (time × group), which represents the treatment effect. Models were fitted using maximum likelihood (Gaussian and binomial outcomes) or Laplace-approximated likelihood (negative-binomial outcomes) as implemented in the lme4 and glmmTMB packages. t- and z-statistics were converted to two-sided p-values. Denominator degrees of freedom for linear mixed models were obtained via Satterthwaite’s approximation (package lmerTest). Model assumptions (normality and homoscedasticity of residuals for linear models, dispersion and zero inflation for count models) were inspected graphically and found not to be violated.

At baseline, every participant met the case definition for SWD, resulting in perfect separation and preventing estimation of a longitudinal mixed-effects logistic model. Consequently, the primary analysis for SWD was limited to a cross-sectional logistic regression at follow-up, with group as the sole predictor. Because no valid baseline model could be fitted, an intention-to-treat analysis (e.g., with last-observation-carried-forward) was not feasible; therefore, the SWD analysis was performed as a complete-case analysis, restricted to participants with non-missing SWD status at follow-up. The four context-specific insomnia composite scores (day shift, evening shift, night shift, and rest days), turnover intention, and the five dimensions of the Swedish Occupational Fatigue Inventory were each examined with linear mixed-effects models containing time, group, and time × group as fixed effects, with a random intercept for participant. The number of correct responses on the knowledge test was treated as an approximately continuous variable and similarly examined with a linear mixed-effects model with a random intercept, fitted with time, group, and time × group as fixed effects. Total sickness-absence days and shift work sleep-related sickness-absence days were over-dispersed count variables. Thus, both were modeled with mixed-effects negative-binomial regression (function glmmTMB), again including time, group, time × group, and a random participant intercept. In additional analysis, sickness absence was dichotomized (none vs. any) and analyzed with mixed-effects logistic regression using the glmer function (lme4 package).

### Missing data and sensitivity considerations

For all outcomes except SWD, mixed-effects models were estimated under the assumption of data missing at random; all available observations contributed information (full-information maximum likelihood). For SWD, complete-case analysis was unavoidable because baseline non-variation precluded imputation strategies. Sensitivity analyses based on last-observation-carried-forward were judged inappropriate, because every participant fulfilled the criteria for SWD at baseline and LOCF would therefore add no new information.

## Results

**Table 2** displays the demographics of the study population. Of the total sample, 86.9% were females (n=173), with a mean age of 39.0 years. The majority (69.8%) were cohabiting/married, and 45.2% had children living at home. Most participants were involved in work schedules that included night work. A total of 10.6% reported use of sleep medication on prescription at baseline (see **Table 2**). There were no statistically significant differences regarding age, sex, or marital status between completers and non-completers (all p >.05).

**Table 2:** Demographics at baseline among the 199 participants in the study.

Variables	Total population	Sleep hygiene advice	Self-help book
	(n=199)	(n=99)	(n=100)
Age (mean years, SD)	39.0 (10.1)	39.5 (9.77)	38.5 (10.45)
Gender (%)			
Male	13.1	13.1	13.0
Female	86.9	86.9	87.0
Married/cohabiting (%)	69.8	70.7	69.0
Children living at home (%)	45.2	50.5	40.0
Work schedule (%)			
Day/evening work	14.1	15.2	13.0
Shift schedule with night work	85.9	84.8	87.0
Sleep medication on prescription last month (%)			
Yes	10.6	11.1	10.1
No	89.4	88.9	90.0
Sleep medication (OTC*) last month (%)			
Yes	8.0	10.1	6.0
No	92.0	89.9	94.0

*Over the counter.

At follow-up, 81.3% of the participants in the self-help book group reported to have read parts of or all the written material they received as compared to 90.3% among the participants in the sleep hygiene group (p=0.13). In the self-help book group, 42.2% had read the whole book, while 77.8% had read the sheet with sleep hygiene advice. A total of 46.9% of the participants in the self-help book group reported that they agreed or totally agreed that they had followed the advice from the book as compared to 54.9% of the participants in the sleep hygiene group (p=0.35). A total of 71.9% of the health care workers in the self-help book group reported that they agreed or totally agreed that they were satisfied with the content of the written material as compared to 54.9% of the participants in the sleep hygiene group (p=0.04).

There was a reduction in prevalence of SWD from baseline to follow-up in both groups (from 100% to 79.0% in the intervention group, p<.001, and from 100% to 83.1% in the control group, p<.001) (see **Table 3**). However, the reduction was not statistically significant between the groups (see **Table 4**). The odds of having SWD in the intervention group were estimated to be 23% lower than in the control group (OR = 0.77, 95% CI: 0.32–1.84, p=0.55), e.g., not statistically significant. No significant time × group interactions were found for any of the insomnia composite scores (day shifts, evening shifts, night shifts and rest days, respectively) (see **Table 4**).

**Table 3:** The effect of sleep hygiene advice and self-help book on the prevalence of shift work disorder, insomnia, knowledge of sleep and circadian rhythms, turnover intention, sickness absence and work-related fatigue among 136 health care workers completing the study.

Variables	Sleep hygiene advice, within effects				Self-help book, within effects			
	Baseline	Follow-up	Statistics (df)*	p-value	Baseline	Follow-up	Statistics (df)*	p-value
Shift work disorder Yes No	71 (100%) 0	59 (83.1%) 12 (16.9%)	3.5	**<0.001**	62 (100.0%) 0	49 (79.0%) 13 (21.0%)	3.6	**<0.001**
Insomnia Day work	42.0%	39.1%	0.5	0.617	42.6%	27.9%	2.3	**0.020**
Insomnia Evening work	37.7%	24.6%	2.0	**0.050**	27.9%	14.5%	2.0	**0.046**
Insomnia Night work	66.7%	66.7%	0.0	1.00	54.1%	54.1%	0.0	1.00
Insomnia Rest days	14.5%	2.9%	2.8	**0.005**	9.8%	3.3%	2.0	**0.046**
Knowledge test - correct answers	7.2 (SD = 1.6)	7.1 (SD = 1.7)	0.6 (67)	0.566	7.5 (SD = 2.0)	8.4 (SD = 2.0)	3.6 (58)	**<0.001**
Turnover Intention	8.0 (SD = 3.6)	8.6 (SD = 4.0)	2.1 (68)	**0.044**	7.6 (SD = 3.5)	7.5 (SD = 3.5)	0.3 (58)	0.797
Sickness absence in days (mean)	6.2 (SD = 12.6)	4.6 (SD = 8.7)	0.9 (68)	0.359	4.1 (SD = 6.1)	4.7 (SD = 12.5)	0.4 (58)	0.711
Sickness absence – work time related sleep problems days (mean)	2.5 (SD = 7.0)	1.9 (SD = 2.4)	1.1 (68)	0.295	1.9 (SD = 4.3)	1.5 (2.2)	1.0 (58)	0.311
SOFI Lack of energy	16.22 (SD = 5.3)	15.30 (SD = 6.0)	1.4 (68)	.173	14.86 (SD = 5.9)	12.29 (SD = 5.8)	3.8 (58)	**<0.001**
SOFI Physical exertion	9.38 (SD = 4.7)	9.48 (SD = 4.8)	0.2 (68)	.847	9.32 (SD = 4.4)	8.76 (SD = 4.7)	1.1 (58)	.263
SOFI Physical discomfort	12.94 (SD = 5.6)	12.54 (SD = 5.8)	0.8 (68)	.408	12.44 (SD = 5.5)	10.88 (SD = 5.1)	3.1 (58)	**.003**
SOFI Lack of motivation	13.52 (SD = 4.7)	13.09 (SD = 5.0)	0.8 (68)	.450	11.68 (SD = 4.7)	9.95 (SD = 4.5)	3.0 (58)	**.004**
SOFI Sleepiness	16.36 (SD = 4.7)	15.58 (SD = 4.9)	1.4 (68)	.166	14.66 (SD = 4.9)	12.49 (SD = 4.9)	4.0 (58)	**<0.001**

*McNemar tests or paired t-test. df=degrees of freedom.

The McNemar tests and paired t-tests (separate analyses for the sleep hygiene group and the self-help book group) are based on n=69 in the sleep hygiene group and n=59 in the self-help book group. Values in bold indicate statistically significant results, with p < .05.

**Table 4:** Interaction effects (time x group) of sleep hygiene advice and self-help book on insomnia, a knowledge test, turnover intention and work-related fatigue measured with mixed-effects models, from baseline to 6-months follow-up.

Variables	Estimate	SE	t	p-value
Insomnia Day work	-0.15	0.08	-1.89	0.061
Insomnia Evening work	-0.03	0.09	-0.35	0.725
Insomnia Night work	-0.01	0.07	-0.19	0.849
Insomnia Rest days	-0.02	0.06	-0.38	0.703
Knowledge test - correct answers	1.15	0.31	3.68	**<0.001**
Turnover Intention	-0.64	0.42	-1.53	0.128
SOFI Lack of energy	-2.27	0.92	-2.48	**0.014**
SOFI Physical exertion	-0.80	0.69	-1.16	0.250
SOFI Physical discomfort	-1.27	0.68	-1.87	0.064
SOFI Lack of motivation	-1.90	0.79	-2.40	**0.018**
SOFI Sleepiness	-1.77	0.76	-2.34	**0.021**

Values in bold indicate statistically significant results, with p < .05.

For the knowledge test, the intervention group demonstrated a significantly greater increase in knowledge scores from baseline to follow-up (from mean 7.5 to 8.4) compared to the control group (from mean 7.2 to 7.1), with a significant time × group interaction (p <.001) (see **Table 4**).

The number of days with sickness absence did not change in any of the groups from baseline to follow-up (see **Table 3**) and did not differ between the intervention group and the control group (IRR = 0.85; 95% CI: 0.44–1.62; p=0.62). Similarly, the number of days with sickness absence due to work-related sleep problems did not change from baseline to follow-up in either group (see **Table 3**) and did not differ between them (IRR = 0.59; 95% CI: 0.24–1.43; p=0.244). In addition, the change in the likelihood of having experienced sickness absence (dichotomized sickness absence yes/no) from baseline to follow-up did not differ between the intervention group and the control group (OR = 0.52; 95% CI: 0.17–1.56; p=0.24).

At baseline, the mean turnover intention score was 7.6 (SD 3.5) in the intervention group and 8.0 (SD 3.6) in the control group, while at follow-up, the mean turnover intention scores were 7.5 (SD 3.5) and 8.6 (SD 4.0) in the intervention group and the control group, respectively. The changes in turnover intention from baseline to follow-up was not significant between the two groups (estimate=-0.64, SE = 0.42, p=0.13).

For lack of energy (p=0.014), lack of motivation (p=0.018), and sleepiness (p=0.021), the time × group interactions were statistically significant, with the intervention group showing a greater reduction in symptoms from baseline to follow-up compared to the control group. For the other two work-related fatigue outcomes, no significant differences in change between groups were observed (see **Table 4**).

## Discussion

The prevalence of SWD was reduced in both the control and intervention groups, with no significant difference between the groups in terms of the proportion of workers with SWD at follow-up. However, the self-help book group demonstrated a significantly greater increase in knowledge about sleep, health and shift work from baseline to follow-up compared to the sleep hygiene advice group. Furthermore, three out of the five work related fatigue dimensions significantly improved in the self-help book group compared to the sleep hygiene advice group. For the insomnia composite scores, turnover intention, and sickness absence, no significant differences between the intervention and the control group were detected.

Our first hypothesis, positing that the self-help book for coping with shift work would reduce the prevalence of SWD and reduce sleep- and sleepiness problems more effectively compared to a sheet of paper with sleep hygiene advice was not supported. Several reasons why the self-help book did not reduce the sleep and sleepiness problems related to shift work more effectively than the sleep hygiene advice are possible. Both groups exhibited a decrease in SWD from baseline to follow-up, but there was no statistical difference between the two, even if the odds of having SWD in the intervention group were estimated to be 23% lower than in the control group. It is important to note that the analysis of this outcome was restricted to the completers with non-missing SWD status at follow-up, which reduced the sample size and statistical power. Another explanation why both groups reduced SWD may be regression-to the mean, which implies that participants tend to improve over time regardless of treatment or intervention (34). For the shift-related insomnia outcomes we did not observe any supportive results favoring the intervention compared to the control group, although there was a near-significant interaction effect in relation to day shifts (p = 0.061). This could indicate a possible benefit that warrants further investigation in larger samples.

Although we collected self-reported information on participants’ engagement with the written material, including whether they read and followed the advice, these measures provide only a partial indication of actual use and cannot capture how consistently or in what manner the recommendations were implemented in daily practice. In addition, it should be acknowledged that changes in the outcome variables may also be attributed to factors such as expectancy, selective reading, receiving a more substantial resource, as well as self-presentation in questionnaire responses. Only 42.2% of the participants in the self-help book group read the whole book, while 39.1% reported to have read parts of the book. We lack information on the extent and specific sections of the book that was read. The book was divided into three parts, the first part included information about basic sleep regulation and circadian rhythms, the second part focusing on negative health consequences of shift work, and the final section consisting of practical advice on how to best cope with shift work. One can speculate that participants needed to read all three parts of the book, and especially the final section to achieve optimal effects. Moreover, the follow-up period of the study was six months, which may be insufficient to detect measurable improvements.

In support of our second hypothesis, the intervention demonstrated a statistically significant effect on the knowledge test. The health care workers in the self-help book condition showed a greater increase in knowledge scores compared to controls, highlighting the effect of the book as a source of relevant knowledge of sleep and shift work–related issues. This finding is important, as knowledge may represent a necessary antecedent to behavioral change which would be in line with the health literacy model by Nutbeam (35), even if immediate clinical outcomes regarding sleep or sleepiness may be absent in the present study. In line with this the authors of a review published in 2013, highlight the effect of educational interventions for shift workers (14).The authors emphasize that shift workers regardless of SWD would benefit from knowledge that encompasses awareness of periods of enhanced vulnerability to performance impairment, evidence-based strategies to sustain alertness, methods to improve sleep quality, and promotion of general health behaviors, such as regular physical activity and sufficient restorative sleep (14). Generally, studies show that self-help books targeting specific problems often lead to significant improvement in knowledge and coping strategies, but that adherence could be challenging (36).

Our third hypothesis included several measures, related to both self-reported sickness absence, turnover intention and work-related fatigue. We found support for three of the dimensions of work-related fatigue, whereas no significant interaction effects were present for sickness absence and turnover intention, thus giving partly support for the third hypothesis. The self-help book group exhibited significant reductions in lack of energy, lack of motivation, and sleepiness compared to the sleep hygiene group. These findings suggest that the book positively influenced subjective experiences of fatigue, which are highly relevant to occupational functioning and well-being (26). Also, the reduction in the fatigue component of sleepiness may demonstrate a benefit of the self-help book targeting one of the core symptoms of SWD. The absence of effects on the other two fatigue dimensions indicates that the benefits may be domain-specific rather than generalized.

Regarding sickness absence, analyses across the multiple operationalizations (the number of sickness absence days, sleep-related sickness absence or dichotomized sickness absence) consistently showed no significant effects of the intervention. These null findings suggest that the intervention did not lead to reductions in absence during the 6-month study period. One possible explanation for this outcome could be that the follow-up duration was insufficient to capture effects on sickness absence, or the possibility that sickness absence may be influenced by other organizational and contextual factors beyond the individual-level interventions. A randomized controlled trial carried out in Australia targeting individual sleep of a shift work education and coaching program to manage SWD in nurses also reported no significant reduction in objectively recorded sick leave between the interventions and the controls with a 6-month follow-up period. However, in that study, the baseline sick leave was lower than the average rate for health care workers, thereby limiting the possibility for further reduction (37).

Shift workers experiencing sleep and sleepiness problems are at greater risk of both turnover intention, but also actual turnover. A previous study among Norwegian nurses found SWD to be positively associated with turnover intention one year later (16). However, our hypothesis regarding turnover intention was not supported, as there was no significant difference in the change between baseline and follow-up between the two groups. Previous research has linked turnover intention to younger age and short work experience (38). However, the health care workers in the present study had a mean age of about 39 years, thus they were not particularly young, and one may assume that they also have long work and shift work experience that could affect this measure. This allies with higher age being highlighted as a protective factor against turnover and turnover intention among nurses (39). In addition, the healthy worker effect may be another possible explanation why levels of turnover intention did not change as expected as nurses who remain in the profession tend to be healthier, more resilient, and more satisfied with their work, while those experiencing severe health problems or dissatisfaction often leave earlier. For example, a longitudinal study of female shift-work nurses found that health problems were strongly associated with both turnover intention and actual turnover, highlighting that those who remain employed represent a healthier subgroup (40).

Some strengths and limitations of the study should be noted. One asset was the randomized controlled trial comparing two different interventions. Second, most measures included in the study were well validated. Also, to strengthen the interpretation of the data we used mixed-effects model analyses that account for individual variability and allow all available observations to contribute information under a missing-at-random assumption. The main limitation is missing data at follow-up. Nevertheless, for outcomes analyzed with mixed-effects models, these models handle missing data relatively well since they use all available observations without requiring complete data from every participant. Parameter estimation is performed using maximum likelihood, which incorporates information from individuals with incomplete data rather than discarding them. This approach may reduce bias and maintain statistical power compared to traditional methods such as listwise deletion. Importantly, mixed-effects models rely on the assumption that data are missing at random. Violations of this assumption could still bias estimates. For SWD, baseline non-variation (100% meeting the case definition) resulted in perfect separation and precluded longitudinal mixed-effects modeling. Therefore, SWD was analyzed cross-sectionally at follow-up using complete cases. Another possible limitation is that the control group also received an intervention in form of sleep hygiene advice. However, the use of active control intervention is regarded as more scientific rigorous than passive controls (e.g., waitlists) in randomized controlled trials, since design helps account for nonspecific effects such as treatment expectations. The comparison of a comprehensive self-help book with a brief informational leaflet limits attribution of effects to the book’s shift-work-specific content. Any observed differences may reflect variation in amount of material provided or engagement rather than content, and the absence of a no-treatment control precludes conclusions about the effectiveness of either intervention relative to no intervention. Also, since the engagement with the intervention was assessed only through self-report, participants’ actual use and implementation of the recommendations could not be objectively verified. Consequently, the specific intervention components driving the observed improvements remain unclear.

Since all outcomes were self-reported, changes in reported outcomes may partly reflect altered symptom appraisal and attribution rather than objective changes in sleep, fatigue, or work functioning. Therefore, the interpretation of the fatigue measures deserves caution. While the intervention was associated with reductions in self-reported lack of energy, lack of motivation, and sleepiness, these outcomes do not necessarily reflect genuine clinical or physiological recovery, nor can it be ruled out that the results reflect reduced subjective fatigue without restoration. As highlighted by Schumann et al. (41), improvements following an intervention may reflect enhanced effort mobilization, that is, an increased willingness or ability to cope with demands, rather than restoration of attentional resources. Accordingly, educational interventions may influence how participants understand and manage fatigue-related symptoms, and the observed improvements may reflect changes in symptom appraisal, coping strategies of self-management rather than recovery processes per se.

A limitation is that participants were recruited through convenience sampling, which may limit the representativeness of the sample and reduce the generalizability of the findings. Finally, the study was solely based on subjective self-report data to assess both main outcomes like SWD, as well as insomnia and sickness absence, and this represents a limitation. Furthermore, the study included only health care workers, and mostly females, limiting the generalizability to other professions and to male workers.

## Conclusion

Overall, the findings in the present study highlight a varied pattern of results. Compared to sleep hygiene advice, the self-help book improved both knowledge about shift work and sleep and reduced several aspects of fatigue. However, no differences between the intervention and control group were seen in SWD prevalence, insomnia, or sickness absence. Consequently, the findings support the potential value of the self-help book as an educational resource for shift workers for some symptoms bothering shift workers. Still the study does not provide evidence that the self-help book is an effective treatment for shift work disorder. Future research should examine participants’ real-world use and implementation of the book’s recommendations, explore longer follow-up periods, and interventions that combine education with behavioral and/or organizational adjustments, including therapist-supported treatments or evaluations of self-help as the lowest step in a stepped-care model, to more effectively address the challenges of shift work. Studies addressing the mechanisms involved are also encouraged.

## Data Availability

The raw data supporting the conclusions of this article will be made available by the authors, without undue reservation.

## References

[1] Kecklund G Axelsson J . Health consequences of shift work and insufficient sleep. BMJ. (2016) 355:i5210. doi: 10.1136/bmj.i5210 27803010

[2] Merkus SL van Drongelen A Holte KA Labriola M Lund T van Mechelen W . The association between shift work and sick leave: a systematic review. Occup Environ Med. (2012) 69:701–12. doi: 10.1136/oemed-2011-100488 22767871 PMC3597215

[3] Gracia P Han W-J Li J . Nonstandard work schedules in cross-national perspective: A study of 29 European countries, 2005-2015. (2021). doi: 10.31235/osf.io/mz53c

[4] Parent-Thirion A BI Cabrita J Vargas O Vermeylen G Wilczynska A . Eurofound (2017), sixth european working conditions survey – overview report 2017. (2017).

[5] Sun Q Ji X Zhou W Liu J . Sleep problems in shift nurses: A brief review and recommendations at both individual and institutional levels. J Nurs Manag. (2019) 27:10–8. doi: 10.1111/jonm.12656 30171641

[6] Vedaa O Harris A Bjorvatn B Waage S Sivertsen B Tucker P . Systematic review of the relationship between quick returns in rotating shift work and health-related outcomes. Ergonomics. (2016) 59:1–14. doi: 10.1080/00140139.2015.1052020 26072668

[7] Di Milia L Waage S Pallesen S Bjorvatn B . Shift work disorder in a random population sample - Prevalence and comorbidities. PloS One. (2013) 8. doi: 10.1371/journal.pone.0055306 23372847 PMC3555931

[8] Waage S Moen BE Pallesen S Eriksen HR Ursin H Akerstedt T . Shift work disorder among oil rig workers in the North Sea. Sleep. (2009) 32:558–65. doi: 10.1093/sleep/32.4.558 19413151 PMC2663659

[9] American Academy of Sleep M . International Classification of Sleep Disorders. 3rd ed. Darien, IL: American Academy of Sleep Medicine (2014).

[10] Pallesen S Bjorvatn B Waage S Harris A Sagoe D . Prevalence of shift work disorder: A systematic review and meta-analysis. Front Psychol. (2021) 12:638252. doi: 10.3389/fpsyg.2021.638252 33833721 PMC8021760

[11] Flo E Pallesen S Mageroy N Moen BE Gronli J Hilde Nordhus I . Shift work disorder in nurses--assessment, prevalence and related health problems. PloS One. (2012) 7:e33981. doi: 10.1371/journal.pone.0033981 22485153 PMC3317447

[12] Wickwire EM Geiger-Brown J Scharf SM Drake CL . Shift work and shift work sleep disorder: Clinical and organizational perspectives. Chest. (2017) 151:1156–72. doi: 10.1016/j.chest.2016.12.007 PMC6859247 28012806

[13] Bjorvatn B Pallesen S Moen BE Waage S Kristoffersen ES . Migraine, tension-type headache and medication-overuse headache in a large population of shift working nurses: a cross-sectional study in Norway. BMJ Open. (2018) 8:e022403. doi: 10.1136/bmjopen-2018-022403 30455385 PMC6252763

[14] Wright KP Bogan RK Wyatt JK . Shift work and the assessment and management of shift work disorder (SWD). Sleep Med Rev. (2013) 17:41–54. doi: 10.1016/j.smrv.2012.02.002 22560640

[15] Waage S Pallesen S Moen BE Bjorvatn B . Restless legs syndrome/Willis-Ekbom disease is prevalent in working nurses, but seems not to be associated with shift work schedules. Front Neurol. (2018) 9:21. doi: 10.3389/fneur.2018.00021 29434568 PMC5796891

[16] Blytt KM Bjorvatn B Moen BE Pallesen S Harris A Waage S . The association between shift work disorder and turnover intention among nurses. BMC Nurs. (2022) 21:143. doi: 10.1186/s12912-022-00928-9 35668393 PMC9169346

[17] Duffield CM Roche MA Homer C Buchan J Dimitrelis S . A comparative review of nurse turnover rates and costs across countries. J Adv Nurs. (2014) 70:2703–12. doi: 10.1111/jan.12483 25052582

[18] D'Ettorre G Pellicani V Greco M Mazzotta M Vullo A . Assessing and managing the shift work disorder in healthcare workers. Med Lav. (2018) 109:144–50. doi: 10.23749/mdl.v109i2.6960 PMC7682180 29701630

[19] Smith MR Eastman CI . Shift work: health, performance and safety problems, traditional countermeasures, and innovative management strategies to reduce circadian misalignment. Nat Sci Sleep. (2012) 4:111–32. doi: 10.2147/nss.s10372 23620685 PMC3630978

[20] Cheng P Drake C . Shift work disorder. Neurol Clin. (2019) 37:563–70. doi: 10.1016/j.ncl.2019.03.003 31256790

[21] van Straten A Cuijpers P . Self-help therapy for insomnia: a meta-analysis. Sleep Med Rev. (2009) 13:61–71. doi: 10.1016/j.smrv.2008.04.006 18952469

[22] Riemann D Espie CA Altena E Arnardottir ES Baglioni C Bassetti CLA . The European Insomnia Guideline: An update on the diagnosis and treatment of insomnia 2023. J Sleep Res. (2023) 32. doi: 10.1111/jsr.14035 38016484

[23] Mimeault V Morin CM . Self-help treatment for insomnia: Bibliotherapy with and without professional guidance. J Consult Clin Psych. (1999) 67:511–9. doi: 10.1037//0022-006x.67.4.511 10450621

[24] Simon L Steinmetz L Feige B Benz F Spiegelhalder K Baumeister H . Comparative efficacy of onsite, digital, and other settings for cognitive behavioral therapy for insomnia: a systematic review and network meta-analysis. Sci Rep. (2023) 13:1929. doi: 10.1038/s41598-023-28853-0 36732610 PMC9894949

[25] Bjorvatn B Lundetrae RS Vedaa Ø Pallesen S Evanger LN . A randomized controlled trial comparing sleep hygiene advice with a self-help book focusing on cognitive behavioral therapy for insomnia: a study among patients with prescribed hypnotics from the GP. Scand J Prim Health Care (2026) 44 (1):1–10. doi: 10.1080/02813432.2025.2525423 40577556 PMC12918340

[26] Bjorvatn B Fiske E Pallesen S . A self-help book is better than sleep hygiene advice for insomnia: a randomized controlled comparative study. Scand J Psychol. (2011) 52:580–5. doi: 10.1111/j.1467-9450.2011.00902.x 21790620

[27] Forthun I Waage S Pallesen S Moen BE Bjorvatn B . A shift to something better? A longitudinal study of work schedule and prescribed sleep medication use in nurses. Occup Environ Med. (2022) 79:752–7. doi: 10.1136/oemed-2022-108251 35725298 PMC9606542

[28] Waage S Pallesen S Moen BE Vedaa O Thun E Vikanes Buchvold H . Changes in work schedule affect the prevalence of shift work disorder among Norwegian nurses - a two year follow-up study. Chronobiol Int. (2021) 38:924–32. doi: 10.1016/j.sleep.2019.11.1131 33736559

[29] Flo E Bjorvatn B Folkard S Moen BE Gronli J Nordhus IH . A reliability and validity study of the bergen shift work sleep questionnaire in nurses working three-shift rotations. Chronobiol Int. (2012) 29:937–46. doi: 10.3109/07420528.2012.699120 22823877

[30] Warpe H Bjorvatn B Waage S Hovlid E Pallesen S . Vet lite om helsefarene ved turnusarbeid. Sykepleien. (2013) 101:54–7. doi: 10.4220/sykepleiens.2013.0104 41213920

[31] Bowling NA Hammond GD . A meta-analytic examination of the construct validity of the Michigan Organizational Assessment Questionnaire Job Satisfaction Subscale. J Vocational Behav. (2008) 73:63–77. doi: 10.1016/j.jvb.2008.01.004 42574925

[32] Ahsberg E Gamberale F Gustafsson K . Perceived fatigue after mental work: an experimental evaluation of a fatigue inventory. Ergonomics. (2000) 43:252–68. doi: 10.1080/001401300184594 10675062

[33] Faul F Erdfelder E Lang AG Buchner A . G*Power 3: a flexible statistical power analysis program for the social, behavioral, and biomedical sciences. Behav Res Methods. (2007) 39:175–91. doi: 10.3758/bf03193146 17695343

[34] Yu R Chen L . The need to control for regression to the mean in social psychology studies. Front Psychol. (2014) 5:1574. doi: 10.3389/fpsyg.2014.01574 25620951 PMC4287101

[35] Nutbeam D . Health literacy as a public health goal: a challenge for contemporary health education and communication strategies into the 21st century. Health Promot Int. (2000) 15:259–67. doi: 10.1093/heapro/15.3.259 40388063

[36] Gürlek TTT Mehmet Hakan . A review about the use and effectiveness of self-help books in psychology. J Cognitive-Behavioral Psychother Res. (2024) 13:331–40. doi: 10.14744/JCBPR.2024.83495

[37] Booker LA Sletten TL Barnes M Alvaro P Collins A Chai-Coetzer CL . The effectiveness of an individualized sleep and shift work education and coaching program to manage shift work disorder in nurses: a randomized controlled trial. J Clin Sleep Med. (2022) 18:1035–45. doi: 10.5664/jcsm.9782 34870586 PMC8974377

[38] Cho SH Lee JY Mark BA Yun SC . Turnover of new graduate nurses in their first job using survival analysis. J Nurs Scholarship. (2012) 44:63–70. doi: 10.1111/j.1547-5069.2011.01428.x 22233430

[39] Hayes LJ O'Brien-Pallas L Duffield C Shamian J Buchan J Hughes F . Nurse turnover: A literature review - An update. Int J Nurs Stud. (2012) 49:887–905. doi: 10.1016/j.ijnurstu.2005.02.007 22019402

[40] Ki J Choi-Kwon S . Health problems, turnover intention, and actual turnover among shift work female nurses: Analyzing data from a prospective longitudinal study. PloS One. (2022) 17. doi: 10.1371/journal.pone.0270958 35802575 PMC9269367

[41] Schumann F Steinborn MB Kurten J Cao L Handel BF Huestegge L . Restoration of attention by rest in a multitasking world: Theory, methodology, and empirical evidence. Front Psychol. (2022) 13:867978. doi: 10.3389/fpsyg.2022.867978 35432083 PMC9010884

